# High prevalence but low referral rate of adrenal incidentalomas in a Lebanese tertiary hospital

**DOI:** 10.3389/fendo.2025.1598685

**Published:** 2025-07-29

**Authors:** Isabelle Jambart, Anne-Marie Wakim, Nadine Cheaib, Christelle Lahoud, Maryam Mansour, Carla Bou Issa, Charbel Daoud, Lina Menassa Moussa, Marie-Hélène Gannagé-Yared, Nada El Ghorayeb

**Affiliations:** ^1^ Department of Endocrinology at Hôtel-Dieu de France Hospital, Faculty of Medicine, Saint-Joseph University, Beirut, Lebanon; ^2^ Department of Radiology at Hôtel-Dieu de France Hospital, Faculty of Medicine, Saint-Joseph University, Beirut, Lebanon

**Keywords:** adrenal incidentaloma, Lebanon, radiological characteristics, prevalence, referral

## Abstract

**Introduction:**

Adrenal incidentalomas (AI) are being more frequently reported with the advent of medical imaging. Studies on their prevalence, characteristics and functional status are lacking in the Middle East.

**Objective:**

To determine the prevalence of AI as well as their clinical and radiological characteristics and referral rates to endocrinologists for hormonal testing.

**Methods:**

We prospectively evaluated unselected adult patients who underwent an abdominal computed tomography (CT) between November 2022 and June 2023 at a Lebanese tertiary teaching hospital. An experienced radiologist carefully reviewed 4,464 CT. Patients with known or suspected malignancy or adrenal disease were excluded. Main outcome measures included prevalence of AI and referral rates to endocrinologists during a one-year follow-up.

**Results:**

3168 CT were retained for analysis, 222 had an AI, with an overall prevalence of 7%. There was no significant difference in prevalence according to gender (8% in women, 6% in men p = .06.). The prevalence increased with age from 0.9% in young adults (18–30 years) to 10.8% in patients older than 70. Mean age of patients with AI was 69 ± 14 years. 46% were males. Median size of AI was 1.7 cm (1.2-2.5). 45% had a density <10 Hounsfield Unit (HU) and 17% were bilateral. Patients with an AI were significantly older (p <.0001), had a higher Body mass index (BMI) (p = .0021), more hypertension (p = .023), dyslipidemia (p = .002), and cardiovascular diseases (CVD) (p = .0056). No difference according to sex was noted. The referral rate of AI to endocrinologists was only 5.4%.

**Conclusion:**

The prevalence of AI in Lebanon is comparable to the worldwide prevalence and is linked to age, BMI, hypertension, dyslipidemia and CVD but not to gender. Despite this high prevalence, the rate of referral for appropriate investigations is poor. Raising awareness among clinicians is therefore essential for a better evaluation of these patients.

## Introduction/background

Recent advances of imaging technologies and the widespread use of high-resolution cross-sectional imaging in medical practice has increased the frequency of detection of adrenal disorders (1). “Adrenal Incidentalomas” (AI) are defined as adrenal masses larger than 1 cm found incidentally on abdominal imaging performed initially for another purpose (2). This definition excludes lesions discovered during cancer staging or suspicion of adrenal diseases (3). The incidence of AI has increased 10-fold during the last two decades (4). This increase parallels the technological advances of imaging modalities. AI are thus considered the pathology of modern technology (1). AI are mainly unilateral and are bilateral in only 10-15% of cases (1, 2). Their prevalence increases with age, from less than 1% in people less than 30 years old in autopsy series to 7% in people more than 70 years old (5). They are particularly more frequent in Caucasians patients with obesity, diabetes, and hypertension (1).

Discovery of an AI raises two questions whether it is malignant or if it is secreting excess amounts of hormones. Imaging and biochemical testing are crucial tools to answer these questions. Malignancy is found in 5 to 8% of adrenal masses while 15 to 20% are functional (5 to 30% for hypercortisolism whether clinical or subclinical, 3-7% for pheochromocytomas, and 1 to 10% for primary aldosteronism (PA)) depending on the studied population (1). This highlights the need to evaluate and appropriately treat these patients because of the potential detrimental consequences and complications (6, 7).

Unenhanced computer tomography (CT) offers a valuable tool for evaluating the lipid content within adrenal masses. For homogeneous lesions, a Hounsfield Unit (HU) value below 10 on a non-contrast CT scan strongly suggests a lipid-rich composition. This finding can be highly reassuring, as it reliably excludes malignancy, even in larger adrenal masses. Only lesions larger than 4 cm or with a density greater than 20 HU are typically managed surgically due to their increased risk of malignancy (7). All other cases should be discussed using a multidisciplinary approach (8), including the referring physician, the endocrinologist, the radiologist, and the surgeon.

To find out if the adrenal lesion is functional, a hormonal workup should be performed for all patients with AI (4, 9). This workup includes excluding hypercortisolism in all patients, pheochromocytomas in AI with a density more than10 HU, and PA in patients with AI and hypertension (4). Moreover, in cases of bilateral adrenal masses, congenital adrenal hyperplasia should be ruled out (4).

To date, studies evaluating the prevalence of AI in the Middle East are lacking (10). Furthermore, reports investigating the rate of referral to endocrinologists are scarce (10–13) Our study aims to determine the prevalence and characteristics of AI in a tertiary care center in Lebanon, as well as the proportion of AI that were appropriately followed for hormonal testing.

## Design and methods

### Study design

We conducted a prospective observational descriptive study. The population consisted of adult patients who performed an abdominal computed tomography (CT) between November 2022 and June 2023 at Hotel-Dieu de France hospital in Lebanon. Exclusion criteria were: age less than 18 years-old, adrenal masses less than 1 cm or adrenal hyperplasia, CT ordered in the context of cancer staging or surveillance, or in the context of suspicion of adrenal disease. For patients who had several CT scans during the same study period, only the first one was included.

### Data collection

#### Radiologic data

The CT scanner used in the study is a GE VCT 64 Light speed. The CT acquisition technical parameters were adjusted according to the clinical needs of the scanner and the patient’s size. The slice thickness is 1.25 mm. A helical scan was performed using 120 kV, 300 mAs, a rotation time of 0.42 seconds, and a pitch of 1.375. An experimented radiologist interpreted all the scans. When an adrenal tumor was discovered, a multiplanar reconstruction of the entire tumor area was performed in the sagittal, coronal, and oblique planes, and the maximum density observed in the mass was reported. The presence and number of adrenal tumors as well as their characteristics (maximal size, density expressed in HU) were collected prospectively from the radiology department. In the case of multiple lesions, the characteristics of the largest lesion were used for analysis. Incidentaloma density was categorized as low (<10 HU), medium (10–20 HU), or high (>20 HU), and size was categorized as small (1–2 cm), medium (2.1–4 cm), or large (>4 cm).

### Demographic and anthropometric characteristics of the population

For hospitalized patients and those admitted to the emergency department, data were collected via the computerized system of the hospital, DxCare. Patients undergoing a CT scan as outpatients were asked to fill a questionnaire that included the following demographic and clinical data: age, sex, presence of hypertension, dyslipidemia, diabetes and cardiovascular diseases (CVD). Body Mass Index (BMI) was calculated using the following formula: weight in kilograms divided by the square of height in meter. Patients were divided into 6 age categories (18-30, 31-40, 41-50, 51-60, 61-70, > 70) in order to evaluate the prevalence according to age.

### Referral rate

Patients’ files were studied between 3 and 12 months after the performance of the CT scan to determine how many of them were referred to an endocrinologist for hormonal investigation.

### Statistical analysis

The variables were entered into an Excel sheet. The statistical analysis was performed using IBM SPSS Statistics software version 26.0 (IBM Corp., Armonk, New York) and GraphPad Prism 8 (La Jolla). Data were analyzed with the Kolmogorov-Smirnov test to determine the variable distribution. Results for continuous variables with a normal distribution were expressed as mean ± SD, and those with non-normal distributions as median (interquartile range). Comparison between groups was analyzed for normally distributed data using Student’s t-test for independent samples and for non-normally distributed data using the Kruskal-Wallis and Mann-Whitney U tests. One-way analysis of variance followed by Bonferroni’s test was used to compare quantitative variables between groups, and the chi-square test or Fisher’s exact test for qualitative variables. The level of significance was set at 5% (p-value less than.05). Multivariable analyses of the predictors for AI were performed using binomial logistic regression analysis and are reported as odds ratios (ORs) with a 95% confidence interval (CI). Predictors with significant p-values in the univariable analysis as well as those with clinical significance related to AI were incorporated in the multivariable model.

### Ethical considerations

The study was approved by the Ethics and Deontology Committee of Saint-Joseph University of Beirut – Lebanon (reference number Tfem/2023/71). All participating patients provided their informed consent by voluntarily completing the study questionnaire. Data collected were subsequently anonymized to ensure patient confidentiality before analysis.

## Results

### Characteristics of the overall population

**Figure 1:**
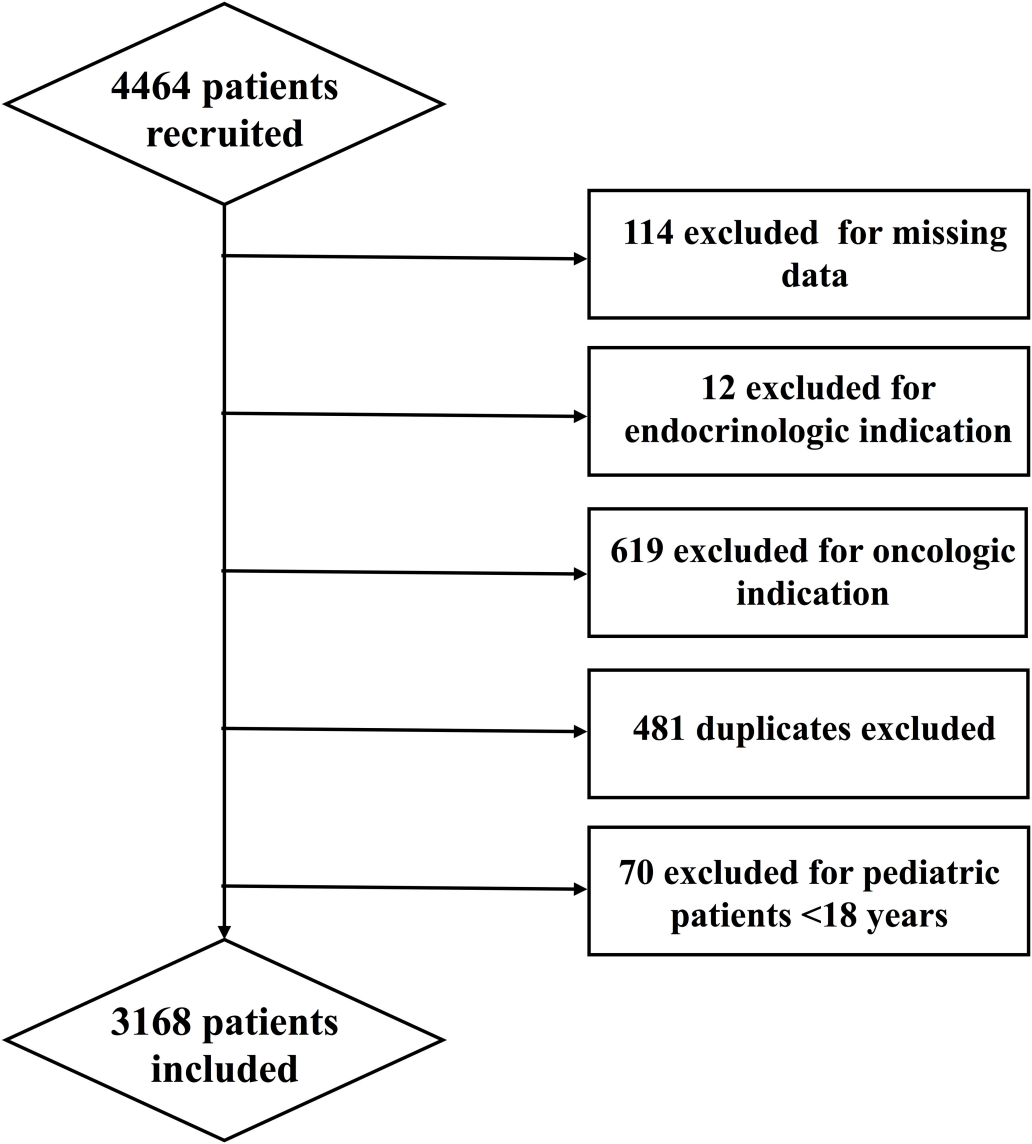
Study flowchart.

Of the 4,464 CT scans analyzed, 1296 were excluded due to missing data or failure to meet inclusion criteria (**Figure 1**). The mean age in our population was 59.76 ± 17.91 years. 53% were men and 47% were women. Mean BMI was 25.8 ± 9.19 kg/m^2^. 48% of our patients had hypertension, 43% had dyslipidemia, 23% had diabetes and 28% had CVD.

### Prevalence of AI according to age, sex, and radiologic characteristics

**Figure 2:**
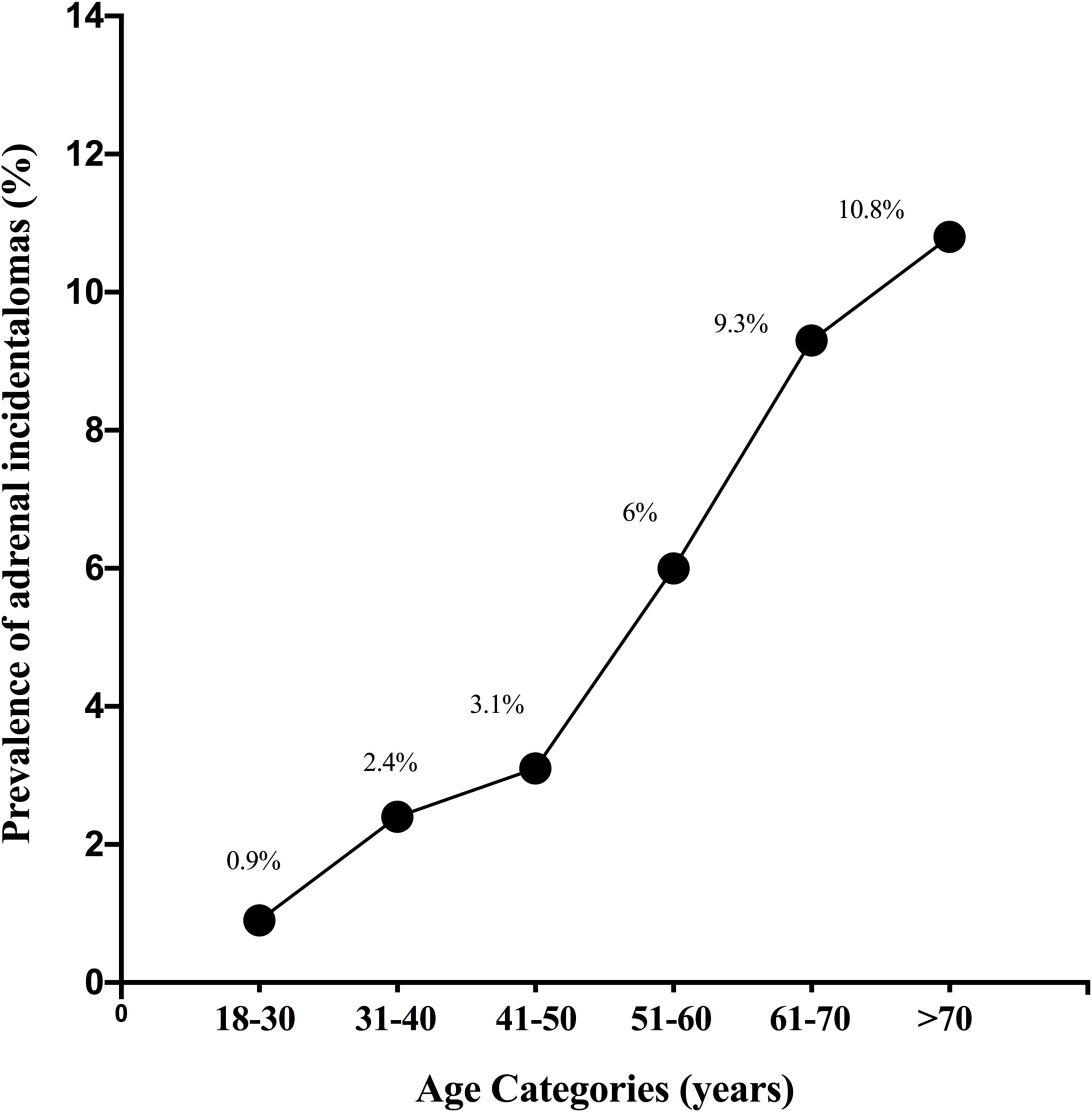
Prevalence of adrenal incidentalomas according to age categories.

Of the remaining 3168 patients, 222 were found to have an AI. The prevalence of AI in our center was therefore 7%. The prevalence was 8% in women and 6% in men with no significant difference between the two groups (p = .06.) The prevalence increased with each age category, from 0.9% in the age group of 18–30 years to 10.8% in patients older than 70 years (p<0.001) (**Figure 2**).

The median size of AI was 1.7 cm (1.2-2.5). 59% of AI had sizes between 1 and 2 cm, 34% were between 2 and 4 cm, and 7% of them were larger than 4 cm. 18% of AI were on the right side, 65% on the left side, and 17% bilateral. 45% of AI had a density lower than 10 HU, 10% between 10 and 20 HU, and 45% had a density higher than 20 HU.

### Comparison of the AI subgroup with the non-AI subgroup

In the group of patients with AI: 46% were men, 54% were women; mean age was 69 ± 14 years; mean BMI was 29.3 ± 16.7 kg/m^2^; 63% had hypertension, 63% had dyslipidemia, 33% had diabetes, and 46% had a history CVD. On the other hand, in the group of patients without AI, 53% were men, 47% are women; mean age was 59 ± 18 years; mean BMI was 25.5 ± 8.3 kg/m^2^; 47% had hypertension; 41% had dyslipidemia; 23% had diabetes; and 27% had a history of CVD. Therefore, patients with AI were significantly older, had a higher BMI, and had more hypertension, dyslipidemia, and CVD than the patients without AI (<. 0001,.0021,.023,.002 and.0056 respectively) (**Table 1**). In the multivariable logistic regression analysis (**Table 2**), body mass index (BMI) emerged as the only independent predictor significantly associated with the presence of AI (OR = 1.107; 95% CI: 1.041–1.178; p = 0.001).

**Table 1:** Comparison between patients and without an adrenal incidentaloma.

Patients characteristics	Patients with an adrenal incidentaloma (N = 223)	Patients without an adrenal incidentaloma (N = 2945)	*P* value
Male Sex (%)	46	53	.322
Age (years ± SD)	69 ± 14	59 ± 18	<.0001
Body Mass Index (kg/m^2^ ± SD)	29.3 ± 16.7	25.5 ± 8.3	.0021
Hypertension (%)	63	47	.023
Dyslipidemia (%)	63	41	.002
Diabetes (%)	33	23	.115
Major cardiovascular events (%)	46	27	.0056

Data are expressed as mean ± SD or as absolute value and percentage as appropriate.

**Table 2:** Multivariable logistic regression model of the predictors the presence of AI.

Predictors	OR (95% CI)	*P* value
Age (years)	1.020 (.994 – 1.046)	.133
Body Mass Index (kg/m^2^)	1.107 (1.041 – 1.178)	.001
Hypertension	.888 (.423 – 1.865)	.754
Dyslipidemia	.564 (.277 – 1.148)	.114
Diabetes	1.937 (.858 – 4.375)	.112
Cardiovascular risk diseases	.798 (.374 – 1.703)	.559

OR, Odds Ratio; CI, Confidence Interval.

### Referral rate and/or investigation for hormonal hypersecretion

After a period of at least 3 months and up to one year, only 12 out of the 222 patients with AI (5.4%) were referred to an endocrinologist or any other specialist for investigation in order to rule out excess hormone secretion.

## Discussion

The prevalence of AI in our population is 7%. The prevalence of AI reported in the literature is extremely variable, ranging from 0.4% to 7.3% (**Table 3**) (10, 14–26) This variability could be attributed to several factors, including the characteristics of the study population (age, health status, geographic location, exclusion criteria), sensitivity of the imaging procedures, nature of the study (retrospective versus prospective studies), and sample size. Overall, our prevalence is one of the highest among published studies, along with the one reported by Reimondo et al. in Italy (7%) (25). The high-resolution of our CT scan and the prospective nature of our study might partly account for this higher prevalence. Indeed, studies have proved that the prevalence of AI increases from approximately 0.64% to 4-5% with the use of more powerful CTs (1, 27). In addition, retrospective studies tend to demonstrate a lower incidence of AI (as low as 2.5%), probably because AI are often not mentioned on the final report (27). Finally, the fact that our hospital is a tertiary center that handles more complex medical conditions might also explains the higher prevalence observed in our study. Notably, we found a high prevalence of AI despite having excluded all oncologic patients. Our prevalence may have been even higher had we included subjects undergoing scans for tumor or metastasis detection.

**Table 3:** Prevalence of adrenal incidentalomas in CT scan series.

Study	Study Period	Number of patients	Age at Diagnosis (years)	Female Sex (%)	Type of CT	Prevalence of Adrenal Incidentalomas (%)	Size (mm)	Bilateral masses (%)
Glazer, 1982 (14)	NA	2200	NA	NA	NA	0.6	NA	NA
Prinz, 1982 (15)	1981	1423	41-73	44.4	Abdominal	0.6	10-40	NA
Abecassis, 1985 (16)	1983-1985	1459	NA	NA	NA	1.3	NA	NA
Belldegrun, 1986 (17)	1976-1983	12 000	NA	NA	Abdominal	0.7	NA	NA
Herrera, 1991 (18)	1985-1989	61 054	62	60.2	NA	0.4	2, (10-110)	NA
Caplan, 1994 (19)	NA	1779	NA	NA	NA	1.90	NA	NA
Song, 2008 (20)	2000-2003	65 231	64 (19-100)	NA	Abdominal and thoracic	1.5	20 (4-82)	7.8
Hammarstedt, 2010 (21)	2002-2004	34 044	69 (30-94)	56.9	Abdominal and thoracic	4.5	25.8 (8-94)	25.1
Bovio, 2006 (22)	2001-2001	520	58 (50-79)	26.1	Thoracic scan	4.4	12-38	13.2
Davenport, 2011 (23)	2006-2007	3099	68 (45-92)	46	Abdominal and thoracic	0.98 abdomen 0.81 thorax	26 ± 12	2.7
Grossman, 2016 (24)	NA	673	50.93 ± 11.1	NA	Abdominal CT	4.2	NA	11
Reimondo et al, 2020 (25)	2017-2018	601	65.6 ± 10.3	27.3	Abdominal CT	7.3	21 (10-50)	29.5
Jing Y et al, 2022 (41)	NA	25 000	NA	NA	Abdominal	1.4	NA	NA
Elzain et al, 2024 (10)	2013 – 2021	300	NA	50.3	Abdominal	NA	NA	4
Present Study	2022-2023	3168	69 ± 14	54	Abdominal and thoracic	7	24 ± 45	17

Range in parentheses indicates the minimum value and the maximum value of the sample.

CT, computed tomography; NA, not available.

We did not find any statistically significant difference in the distribution of AI between men and women. In the literature, AI were found to be more prevalent in women in several radiological series (1, 18, 21, 28). Conversely, two published studies demonstrate a male predominance (25, 29). Male sex has also been associated with a greater risk of malignancy (30). Another study showed that patients with AI frequently develop hypertension and hyperglycemia regardless of sex (31).

Our study showed the expected increase in the prevalence of AI with age. This is in accordance with all previously published studies (1, 32–34) Increased adrenal tumorigenesis with age is likely driven by both an increase in genetic mutations as well as remodeling of the tissue microenvironment (35). Increased use of imaging in older subjects could also explain their higher prevalence of AI.

The median size of AI was 1.7 cm (1.2-2.5), with the majority (59%) ranging between 1 and 2 cm, and most of them (83%) being unilateral, mainly located on the left side (65%). This is similar to the findings in the literature, with studies reporting an average size of 3 cm, with a range of 0.8 to 2.3 cm, and left sided predominance (27, 29, 36, 37) The predominance on the left size might be due to a detection bias, since left-sided adenomas are more easily detectable to radiologists. Indeed, several CT series have shown an equal distribution between the right and left adrenal glands, similarly to autopsy series (27, 29, 36). Furthermore. The density of our incidentalomas is mostly either lower than 10 HU (45%) or higher than 20 HU (45%). However, in a large retrospective study conducted by Ebbehoj et al., the majority of AI had a density inferior to 10 HU (54.7%), and only 21.6% had a density of 20 or more (38). The high proportion of AI with a density > 20 HU in our study underlies the importance of recognizing and investigating AI and could be explained by the fact that our patients were recruited from a tertiary care hospital, with most of them being possibly more ill than patients in primary care settings.

Moreover, AI in our study are significantly more frequent in patients with a higher BMI, hypertension, dyslipidemia, and a history of CVD. According to our multivariable analysis, however, the only statistically significant clinical predictive factor for AI was BMI. This finding suggests that, after adjusting for potential confounders, each one unit increase in BMI is associated with a 10.7% increase in the odds of having an AI. In the study published by Reimondo et al., higher BMI was also significantly associated with the presence of AI, but hypertension, dyslipidemia and CVD were not. Type 2 diabetes however, was significantly more frequent in the group with AI (25) in contrast to our study where diabetes was frequent but didn’t reach statistical significance. This is most probably because patients with autonomous cortisol secretion were not included in our study. The association between AI and the presence of co-morbidities is mainly due to the fact that some AI are functional and secrete either cortisol or aldosterone or catecholamines, which highlights the importance of investigating all AI.

Finally, the referral rate of AI to endocrinology in our study is very low (5.4%). The few published studies analyzing this issue also revealed that clinicians rarely prescribe the appropriate medical assessments and/or refer their patients to an endocrinologist (11, 12). Indeed, a study conducted in a large UK university teaching hospital reported that only 9.6% of patients with AI were referred to an endocrinologist (11). Another review of 804 AI showed that 30% were investigated and followed-up (13). Our study has the lowest referral rate, even though 45% of our AI have a density higher than 20 HU and 7% are larger than 4 cm. Lebanon’s ongoing political and financial crisis might contribute to this lower referral rate, as it could deter healthcare providers from pursuing investigations and many cases were lost to follow-up. Keeping in mind that 37% of AI might increase in size on follow-up (39), and 29% of patients with nonfunctioning AI could develop mild autonomous cortisol secretion during follow-up (40), AI should be followed-up regularly by an endocrinologist to rule out a change in size or aspect on imaging or a new hormonal secretion. The European Society of Endocrinology clinical practice guidelines state that patients with an adrenal incidentaloma should undergo a 1mg overnight dexamethasone suppression test to rule out hypercortisolism. They also recommend no further imaging for benign appearing adrenal masses (homogeneous appearance and a density ≤ 10 HU). As for non-secreting homogenous adrenal masses with a size inferior to 4 cm and a density of 11–20 HU, they suggest either an immediate additional imaging or a follow-up CT scan or MRI in 12 months. To improve patient outcomes, it is therefore essential to raise awareness among physicians of all specialties about the significance of evaluating and monitoring AI, in line with the latest European Society of Endocrinology guidelines (7).

The large sample size of 3168 patients increases the accuracy of our results as well as the validity of our conclusions. Moreover, the prospective design of this study minimizes the likelihood of missed diagnoses of AI by the radiologist. Furthermore, our study is the first conducted in Lebanon and the second in the Middle East regarding the prevalence, clinical and radiological characteristics, and referral rate of AI.

Nevertheless, some limitations should be considered. Patients were selected from a single center, which could induce a selection bias and limit the generalizability of our results. Additionally, the fact that every oncologic patient was excluded might underestimate the prevalence of AI in our population. Moreover, our data collection relies on medical records and patient questionnaires, which may be subject to missing or inaccurate information. Finally, it is important to remember that the evolving nature of imaging technology can influence the detection of AIs, necessitating a continuous reevaluation of findings.

## Conclusion

Our study revealed a prevalence of AI of 7%. AI were found to be more frequent in older people as well as patients with higher BMI, hypertension, dyslipidemia, and a history of CVD. The very low referral rate to endocrinologists in our study may be attributed to Lebanon’s ongoing crisis and developing healthcare system. To optimize patient outcomes, raising awareness among healthcare providers about the significance of AI and the need for timely referral is crucial.

## Data Availability

The original contributions presented in the study are included in the article/supplementary material. Further inquiries can be directed to the corresponding author.

## References

[1] Chatzellis E Kaltsas G. Adrenal incidentaloma. In: Feingold KR Anawalt B Blackman MR Boyce A Chrousos G Corpas E, editors. Endotext. MDText.com, Inc, South Dartmouth (MA (2000). Available online at: http://www.ncbi.nlm.nih.gov/books/NBK279021/., PMID:

[2] Young WF. Clinical practice. The incidentally discovered adrenal mass. N Engl J Med. (2007) 356:601–10. doi: 10.1056/NEJMcp065470, PMID: 17287480

[3] Farrugia FA Martikos G Surgeon C Tzanetis P Misiakos E Zavras N. Radiology of the adrenal incidentalomas. Review of the literature. Endocrine regulations. (2017) 51. 10.1515/enr-2017-0005 28222025

[4] Bancos I Prete A. Approach to the patient with adrenal incidentaloma. J Clin Endocrinol Metab. (2021) 106:3331–53. doi: 10.1210/clinem/dgab512, PMID: 34260734 PMC8530736

[5] Gaillard Stéphanie Meyer P. The adrenal incidentaloma: disease of modern era. Rev medicale suisse. (2009) 5. doi: 10.53738/REVMED.2009.5.198.0774, PMID: 19418979

[6] Hadjikyriacou E Egan R. Adrenal incidentalomas. Br J Surg. (2022) 109. doi: 10.1093/bjs/znac138, PMID: 35639611

[7] Fassnacht M Tsagarakis S Terzolo M Tabarin A Sahdev A Newell-Price J. European Society of Endocrinology clinical practice guidelines on the management of adrenal incidentalomas, in collaboration with the European Network for the Study of Adrenal Tumors. Eur J Endocrinol. (2023) 189:G1–42. doi: 10.1093/ejendo/lvad066, PMID: 37318239

[8] Ss P Jh K. Recent updates on the management of adrenal incidentalomas. Endocrinol Metab (Seoul Korea). (2023) 38., PMID: 37583083 10.3803/EnM.2023.1779 PMC10475962

[9] Fassnacht M Arlt W Bancos I Dralle H Newell-Price J Sahdev A. Management of adrenal incidentalomas: European Society of Endocrinology Clinical Practice Guideline in collaboration with the European Network for the Study of Adrenal Tumors. Eur J Endocrinol. (2016) 175:G1–34. doi: 10.1530/EJE-16-0467, PMID: 27390021

[10] Elzain W Alshalaan A Qahtani M Qahtani H Assiri D Alshehri H. Adrenal incidentaloma prevalence and clinical management- A retrospective study. Am J Med Sci Innovation. (2024) 3:8–17. doi: 10.54536/ajmsi.v3i1.2332

[11] Hanna FWF Hancock S George C Clark A Sim J Issa BG. Adrenal incidentaloma: prevalence and referral patterns from routine practice in a large UK university teaching hospital. J Endocrine Soc. (2021) 6. doi: 10.1210/jendso/bvab180, PMID: 34988349 PMC8694520

[12] Osborne L Peacey SR. Are adrenal incidentalomas routinely referred to endocrinology services? An audit of referral pattern and appropriate investigation. Endocrine Abstracts. (2010) 21.

[13] Maher DI Williams E Grodski S Serpell JW Lee JC. Adrenal incidentaloma follow-up is influenced by patient, radiologic, and medical provider factors: A review of 804 cases. Surgery. (2018) 164:1360–5. doi: 10.1016/j.surg.2018.07.011, PMID: 30170818

[14] Glazer HS Weyman PJ Sagel SS Levitt RG McClennan BL. Nonfunctioning adrenal masses: incidental discovery on computed tomography. AJR Am J Roentgenol. (1982) 139:81–5. doi: 10.2214/ajr.139.1.81, PMID: 6979870

[15] Prinz RA Brooks MH Churchill R Graner JL Lawrence AM Paloyan E. Incidental asymptomatic adrenal masses detected by computed tomographic scanning. Is operation required? JAMA. (1982) 248:701–4. doi: 10.1001/jama.1982.03330060041031, PMID: 7097921

[16] Abecassis M McLoughlin MJ Langer B Kudlow JE. Serendipitous adrenal masses: prevalence, significance, and management. Am J Surg. (1985) 149:783–8. doi: 10.1016/S0002-9610(85)80186-0, PMID: 4014556

[17] Belldegrun A Hussain S Seltzer SE Loughlin KR Gittes RF Richie JP. Incidentally discovered mass of the adrenal gland. Surg Gynecol Obstet. (1986) 163:203–8., PMID: 3750174

[18] Herrera MF Grant CS van Heerden JA Sheedy PF Ilstrup DM. Incidentally discovered adrenal tumors: an institutional perspective. Surgery. (1991) 110:1014–21., PMID: 1745970

[19] Caplan RH Strutt PJ Wickus GG. Subclinical hormone secretion by incidentally discovered adrenal masses. Arch Surg. (1994) 129:291–6. doi: 10.1001/archsurg.1994.01420270067016, PMID: 8129606

[20] Song JH Chaudhry FS Mayo-Smith WW. The incidental adrenal mass on CT: prevalence of adrenal disease in 1,049 consecutive adrenal masses in patients with no known Malignancy. AJR Am J Roentgenol. (2008) 190:1163–8. doi: 10.2214/AJR.07.2799, PMID: 18430826

[21] Hammarstedt L Muth A Wängberg B Björneld L Sigurjónsdóttir HA Götherström G. Adrenal lesion frequency: A prospective, cross-sectional CT study in a defined region, including systematic re-evaluation. Acta Radiol. (2010) 51:1149–56. doi: 10.3109/02841851.2010.516016, PMID: 20969508

[22] Bovio S Cataldi A Reimondo G Sperone P Novello S Berruti A. Prevalence of adrenal incidentaloma in a contemporary computerized tomography series. J Endocrinol Invest. (2006) 29:298–302. doi: 10.1007/BF03344099, PMID: 16699294

[23] Davenport C Liew A Doherty B Win HHN Misran H Hanna S. The prevalence of adrenal incidentaloma in routine clinical practice. Endocrine. (2011) 40:80–3. doi: 10.1007/s12020-011-9445-6, PMID: 21547511

[24] Grossman A Koren R Tirosh A Michowiz R Shohat Z Rahamimov R. Prevalence and clinical characteristics of adrenal incidentalomas in potential kidney donors. Endocr Res. (2016) 41:98–102. doi: 10.3109/07435800.2015.1076455, PMID: 26541634

[25] Reimondo G Castellano E Grosso M Priotto R Puglisi S Pia A. Adrenal incidentalomas are tied to increased risk of diabetes: findings from a prospective study. J Clin Endocrinol Metab. (2020) 105:dgz284. doi: 10.1210/clinem/dgz284, PMID: 31900474

[26] Prevalence of adrenal incidentaloma in relatively healthy people (2024). Available online at: https://www.jwatch.org/na55334/2022/09/29/prevalence-adrenal-incidentaloma-relatively-healthy-people.

[27] Sherlock M Scarsbrook A Abbas A Fraser S Limumpornpetch P Dineen R. Adrenal incidentaloma. Endocrine Rev. (2020) 41:775. doi: 10.1210/endrev/bnaa008, PMID: 32266384 PMC7431180

[28] Mantero F Terzolo M Arnaldi G Osella G Masini AM Alì A. A survey on adrenal incidentaloma in Italy. Study Group on Adrenal Tumors of the Italian Society of Endocrinology. J Clin Endocrinol Metab. (2000) 85:637–44., PMID: 10690869 10.1210/jcem.85.2.6372

[29] Ahn SH Kim JH Baek SH Kim H Cho YY Suh S. Characteristics of adrenal incidentalomas in a large, prospective computed tomography-based multicenter study: the COAR study in Korea. Yonsei Med J. (2018) 59:501. doi: 10.3349/ymj.2018.59.4.501, PMID: 29749133 PMC5949292

[30] Iñiguez-Ariza NM Kohlenberg JD Delivanis DA Hartman RP Dean DS Thomas MA. Clinical, biochemical, and radiological characteristics of a single-center retrospective cohort of 705 large adrenal tumors. Mayo Clin Proc Innov Qual Outcomes. (2018) 2:30–9. doi: 10.1016/j.mayocpiqo.2017.11.002, PMID: 30225430 PMC6124341

[31] Puglisi S Barač Nekić A Morelli V Alessi Y Fosci M Pani A. Are comorbidities of patients with adrenal incidentaloma tied to sex? Front Endocrinol (Lausanne). (2024) 15:1385808., PMID: 38808113 10.3389/fendo.2024.1385808 PMC11130385

[32] Sconfienza E Tetti M Forestiero V Veglio F Mulatero P Monticone S. Prevalence of functioning adrenal incidentalomas: A systematic review and meta-analysis. J Clin Endocrinol Metab. (2023) 108:1813–23. doi: 10.1210/clinem/dgad044, PMID: 36718682

[33] Young WF Jr. Management approaches to adrenal incidentalomas. A view from Rochester, Minnesota. Endocrinol Metab Clinics North America. (2000) 29. doi: 10.1016/S0889-8529(05)70122-5, PMID: 10732270

[34] Kloos RT Gross MD Francis IR Korobkin M Shapiro B. Incidentally discovered adrenal masses. Endocrine Rev. (1995) 16. 10.1210/edrv-16-4-460 8521790

[35] Warde KM Smith LJ Basham KJ. Age-related changes in the adrenal cortex: insights and implications. J Endocrine Soc. (2023) 7. doi: 10.1210/jendso/bvad097, PMID: 37564884 PMC10410302

[36] Kim J Bae KH Choi YK Jeong JiY Park KG Kim JG. Clinical characteristics for 348 patients with adrenal incidentaloma. Endocrinol Metab (Seoul Korea). (2013) 28. doi: 10.3803/EnM.2013.28.1.20, PMID: 24396646 PMC3811797

[37] Sangwaiya MJ Boland GWL Cronin CG Blake MA Halpern EF Hahn PF. Incidental adrenal lesions: accuracy of characterization with contrast-enhanced washout multidetector CT–10-minute delayed imaging protocol revisited in a large patient cohort. Radiology. (2010) 256. doi: 10.1148/radiol.10091386, PMID: 20656838

[38] Ebbehoj A Li D Kaur RJ Zhang C Singh S Li T. Epidemiology of adrenal tumors - a population-based study in Olmsted county, Minnesota. Lancet Diabetes endocrinol. (2020) 8:894. doi: 10.1016/S2213-8587(20)30314-4, PMID: 33065059 PMC7601441

[39] Jackson BS. Adrenal incidentaloma controversial size recommendations. Urol Res Pract. (2023) 49:96–9. doi: 10.5152/tud.2023.22245, PMID: 37877855 PMC10192718

[40] Petramala L Circosta F Marino L Palombi E Costanzo ML Servello A. Clinical evaluation of adrenal incidentaloma: the experience of a referral center. Biomedicines. (2024) 12:1910. doi: 10.3390/biomedicines12081910, PMID: 39200374 PMC11351527

[41] Jing Y Hu J Luo R Mao Y Luo Z Zhang M. Prevalence and characteristics of adrenal tumors in an unselected screening population : A cross-sectional study. Ann Internal Med. (2022) 175. doi: 10.7326/M22-1619, PMID: 36095315

